# Supplementary material for: Interactions between the transcription factors FfmA and AtrR are required to properly regulate gene expression in the fungus Aspergillus fumigatus
Source: G3 (Bethesda). 2023 Jul 31;13(10):jkad173. doi: 10.1093/g3journal/jkad173 (PMC10542180; doi:10.1093/g3journal/jkad173)
Supplement: jkad173_Supplementary_Data [file jkad173_supplementary_data.zip › Supplementary Table Legends.docx]

Supplementary Table Legends

Supplementary table S1. **Significantly differentially regulated genes in dox-off-*ffmA* cells in the absence of doxycycline.** Genes exhibiting at least a two-fold change and showing P value of <0.05 in DO-*ffmA* cells grown in the absence of doxycycline compared to wild-type are shown.

Supplementary table S2. **Funcat classification of genes showing significant enrichment using FungiFun2 from DO-*ffmA* cells grown without doxycycline**. Enriched genes were only found in the group of genes down by two-fold in DO-*ffmA* cells grown in the absence of doxycycline.

Supplementary table S3. **Significantly differentially regulated genes in dox-off-ffmA cells in the presence of doxycycline.** Genes exhibiting at least a log2=1 change and showing P value of <0.05 in DO-*ffmA* cells grown in the presence of doxycycline compared to wild-type are shown

Supplementary table S4. **Funcat classification of genes showing significant enrichment using FungiFun2 from DO-*ffmA* cells grown with doxycycline**. Enriched genes found with log2=-1 genes in DO-*ffmA* cells grown in the presence of doxycycline.

Supplementary table S5. **Summary of genes that change similarly or differently between *ffmAΔ* or DO-*ffmA*+doxycycline conditions.** Genes that significantly change in data strains lacking ffmA or in the DO-ffmA strain grown in the presence of doxycycline are summarized.

Supplementary table S6. **ChIP-seq peaks common to ChIP reactions performed with either anti-FfmA or anti-FLAG antisera.** ChIP-seq peaks were collected from reactions on wild-type strains using anti-FfmA antibody or on FLAG-FfmA-expressing strain using anti-FLAG antibody. The union of these two data sets is reported.

Supplementary table S7. **Direct target genes enriched via Funcat term analysis using FungiFun2.** Genes detected as common FfmA targets in ChIP-seq data above and also induced in DO-ffmA cells treated with doxycycline are listed.

Supplementary table S8. **Transcriptional proteins enriched via GO term analysis using FungiDB.** Genes detected as common FfmA targets in ChIP-seq data above and also as AtrR targets were analyzed using FungiDB and the transcription factors enriched are listed.

Supplementary table S9. **Asexual sporulation-related proteins enriched via GO term analysis using FungiDB.** Genes detected as common FfmA targets in ChIP-seq data above and also as AtrR targets were analyzed using FungiDB and the genes involved in asexual sporulation are listed.
